# Research Advances on Therapeutic Approaches to Congenital Central Hypoventilation Syndrome (CCHS)

**DOI:** 10.3389/fnins.2020.615666

**Published:** 2021-01-12

**Authors:** Simona Di Lascio, Roberta Benfante, Silvia Cardani, Diego Fornasari

**Affiliations:** ^1^Department of Medical Biotechnology and Translational Medicine (BIOMETRA), Università degli Studi di Milano, Milan, Italy; ^2^CNR–Institute of Neuroscience, Milan, Italy; ^3^NeuroMi–Milan Center for Neuroscience, University of Milano Bicocca, Milan, Italy

**Keywords:** autonomic dysregulation, breathing disorder, congenital central hypoventilation syndrome, CCHS, PHOX2B, polyalanine expansions, desogestrel

## Abstract

Congenital central hypoventilation syndrome (CCHS) is a genetic disorder of neurodevelopment, with an autosomal dominant transmission, caused by heterozygous mutations in the *PHOX2B* gene. CCHS is a rare disorder characterized by hypoventilation due to the failure of autonomic control of breathing. Until now no curative treatment has been found. PHOX2B is a transcription factor that plays a crucial role in the development (and maintenance) of the autonomic nervous system, and in particular the neuronal structures involved in respiratory reflexes. The underlying pathogenetic mechanism is still unclear, although studies *in vivo* and in CCHS patients indicate that some neuronal structures may be damaged. Moreover, *in vitro* experimental data suggest that transcriptional dysregulation and protein misfolding may be key pathogenic mechanisms. This review summarizes latest researches that improved the comprehension of the molecular pathogenetic mechanisms responsible for CCHS and discusses the search for therapeutic intervention in light of the current knowledge about *PHOX2B* function.

## Introduction

Sudden infant death syndrome (SIDS), Rett syndrome (RTT), and congenital central hypoventilation syndrome (CCHS) are human diseases that share developmental defects in the neural circuits that control breathing (Box 1) (Ramirez et al., 2018, 2020; Trang et al., 2020). Breathing is under the control of three linked networks of brainstem neurons responsible for respiratory rhythm generation (Benarroch, 2018, and references therein): the pre-Bötzinger complex (pre-BötC), that drives inspiration, the retrotrapezoid/parafacial respiratory group (RTN/pFRG), that controls active expiration and plays an important role in central chemosensitivity (Guyenet et al., 2019; Pisanski and Pagliardini, 2019), and the post-inspiratory complex (PiCo) controlling post-inspiration (Anderson et al., 2016). The RTN contains glutamatergic neurons, expressing *VGlut2, NK1R, Neuromedin B*, and *PACAP* (Guyenet et al., 2019), whose development depends on the transcription factor paired-like homeobox 2B (PHOX2B), a master gene of the development of the autonomic nervous system (ANS) (Pattyn et al., 1999). Other neural structures are under the developmental control of PHOX2B, such as the locus coerules (LC), the catecholaminergic neurons of the nucleus of solitary tract (NTS) and of the C1 groups, and all these neurons are also activated by hypercapnia, and participate in respiratory reflex (Brunet and Pattyn, 2002; Stornetta et al., 2006; Gargaglioni et al., 2010).

Congenital central hypoventilation syndrome (CCHS or Ondine’s curse; MIM# 209880) is a rare neurological disorder characterized by the deficient autonomic control of breathing (Weese-Mayer et al., 2010; Trang et al., 2020). So far, although some drugs have shown some effects in improving ventilation (Straus et al., 2010; Schirwani et al., 2017), CCHS remains a severe breathing disorder without any effective pharmacological treatment.

*In vitro* and *in vivo* studies have contributed to the elucidation of the physiological role of the *PHOX2B* gene and the molecular consequences caused by its mutations (Di Lascio et al., 2018a). Despite an incomplete picture of the CCHS pathogenetic mechanisms, recent studies have suggested that the development of new therapeutic strategies may target the primary pathogenetic defect of CCHS, or by-pass it by pharmacological compensation. Moreover, the report of ventilation improvement in two CCHS patients using the progestin drug desogestrel as a contraceptive (Straus et al., 2010) has opened up new perspectives for therapeutic research.

In this review we will summarize the current knowledge about the molecular pathogenesis of CCHS and will discuss the recent progress and perspectives in the advancement of therapeutic research in the light of the new insights into the role of PHOX2B.

## Congenital Central Hypoventilation Syndrome (CCHS): Clinical Presentation, Diagnosis, and Management

Alveolar hypoventilation is the hallmark of the disease, caused by an abnormally reduced or absent ventilatory response to hypoxia and hypercapnia caused by the malfunctioning of PHOX2B-mediated regulation of autonomic respiratory control and chemosensitivity. Hypoventilation is generally more severe during sleep [especially during non-rapid eye movement (NREM) sleep] than during wakefulness (Weese-Mayer et al., 2010; Trang et al., 2020). Drugs that stimulate ventilation that have been tested so far are not effective (Weese-Mayer et al., 2010), and management is supported by lifetime assisted ventilation.

The CCHS-defining gene *PHOX2B* (Amiel et al., 2003; Weese-Mayer et al., 2003) encodes a transcription factor whose role as master gene in the development of ANS and of the neural structures involved in breathing control has been clearly defined (Pattyn et al., 1999, 2000). Mutations in the *PHOX2B* gene interfere with the development of the neuronal network that regulates CO_2_ chemosensitivity and breathing (Dubreuil et al., 2008).

The age of presentation of CCHS is typically in newborns, but it may also be diagnosed later in childhood or adulthood [later-onset (LO) CCHS] (Weese-Mayer et al., 2005; Antic et al., 2006; Trochet et al., 2008b; Basu et al., 2017). The disease can be isolated (Di Lascio et al., 2018b; Bachetti and Ceccherini, 2020), or associated with a spectrum of non-respiratory symptoms, among which seizures (Binmanee et al., 2020) and other conditions that reflect a more global ANS dysfuntion, including cardiac arrhythmias and congenital heart disease (Lombardo et al., 2018; Laifman et al., 2020), ocular disorders, the aganglionic megacolon Hirschsprung’s disease and neural crest tumors (reviewed in Di Lascio et al., 2018a,b; Broch et al., 2019; Bachetti and Ceccherini, 2020; Trang et al., 2020).

CCHS is rare: its estimated incidence is 1/148,000–1/200,000 live births (Trang et al., 2005; Shimokaze et al., 2015), with a total of about 1,300 genetically confirmed cases worldwide (Di Lascio et al., 2018a).

Trasmission of CCHS is autosomal dominant with variable expressivity and incomplete penetrance, and without any gender preference (Weese-Mayer et al., 2003; Bachetti and Ceccherini, 2020). Variable CCHS-like phenotypes have been reported associated with whole and partial gene deletions of *PHOX2B* as extensively reported (Jennings et al., 2012; Bachetti and Ceccherini, 2020).

The primary aim of CCHS management is to provide adequate ventilation and oxygenation, and three types of long-life ventilatory support [pressure-controlled ventilation via tracheostomy, non-invasive positive pressure ventilation (mask ventilation), or diaphragm pacing] are commonly used by CCHS patients, according to a number of factors, including the patient’s age, the age of disease onset and ventilatory parameters. The pros and cons of the use of different ventilatory supports are well summarized in the recent published guidelines for diagnosis and management of CCHS (Trang et al., 2020). It is worth noting that the use of some of these supports always requires the presence of carers that monitor the correctness of ventilation and can change the settings, if required, with a great effort of the patient’s families. Moreover, the management of patients with CCHS and the associated conditions of autonomic dysregulation can be more complex, and should include a global assessment of the digestive, cardiovascular, and ocular systems, and the investigation for tumors of neural crest origin (Weese-Mayer et al., 2017; Bishara et al., 2018; Di Lascio et al., 2018a; Maloney et al., 2018; Zaidi et al., 2018; Trang et al., 2020).

Neurocognitive deficits have been reported in CCHS children (Charnay et al., 2016; Zelko et al., 2018), and probably both the primary developmental damage and the chronic episodes of hypoxia–reoxygenation, potentially occurring during nocturnal assisted ventilation but also during wakefulness, contribute to neurological outcome. It has recently been reported that these conditions may cause the overproduction of reactive oxygen species (ROS) (Degl’Innocenti et al., 2018), inducing oxidative stress, cellular damage and eventually activating apoptotic pathway (Rahal et al., 2014). This effect has been already demonstrated in other breathing disorders characterized by intermittent hypoxia such as sudden infant death syndrome (SIDS), obstructive sleep apnea syndrome (OSAS), and Rett syndrome (Prandota, 2004; Lavie, 2015; Bebensee et al., 2017). According to the American Thoracic Society (ATS) recommendations, neuropsychological status should be assessed in all CCHS patients in order to early identify and treat those individuals that would benefit from early interventions to ameliorate their cognitive abilities (Macdonald et al., 2020).

**Box 1.SIDS, RETT, and CCHS main features.**

Sudden Infant Death Syndrome (SIDS)

– SIDS is a complex heterogeneous disorder referred to death during sleep in seemingly healthy infants < 1 year old occurring suddenly and unexplained.
– It is mainly due to respiratory failure during sleep (arouse in response to altered oxygen or CO_2_ levels), and abnormalities in a number of physiological functions and systems (neurological, cardiovascular, respiratory, gastrointestinal, endocrine, metabolic, immune and genetic) have also been reported.
– Its occurrence is estimated in 0.40 SIDS deaths per 1,000 live births.
– No diagnostic features are available up today. SIDS is due to a variety of factors conceptualized in the Triple Risk Model: 1. A critical developmental period in homeostatic control (Central nervous system and Immune system); 2. Vulnerable infant (race or exposure to alcohol or tobacco during pregnancy, genetic polymorphisms, low birth weight, prematurity). 3. Exogenous stressor(s) (overheating, tobacco smoke, upper respiratory tract infection, bed sharing, and prone sleeping position). Death occurs when they are expressed simultaneously.
– One of the main hypothesis is that SIDS is associated with defects in medullary homeostatic control (”brainstem hypothesis”), characterized by malfunctioning neurotransmitter networks, including catecholamines, neuropeptides, serotonin and its receptors, glutamate, brain-derived neurotropic growth factor (BDNF), and some cytokine systems. In particular, abnormalities in the main serotonergic centers [raphe, extra raphe, and ventral (arcuate) populations of 5-HT neurons] and their projection sites (dorsal motor nucleus of the vagus and the nucleus of the solitary tract) and lower expression of brainstem 5-HT, tryptophan hydroxylase-2 (TPH-2), and 5-HT receptor have been reported in SIDS.

RETT Syndrome

– RETT syndrome is a X-linked neurological disorder due to mutation in the *MeCP2* gene, whose symptoms appear 5-18 months after birth.
– Its incidence is 1/10,000 of female births.
– RETT girls show developmental delay, motor impairment, sleep problems, seizures, breathing and feeding dysfunction.
– Breathing dysfunction during wakefulness includes episodes of hyperventilation and irregular breathing and episodes of breath-holding. Respiratory disturbance during sleep includes episodes of obstructive sleep apneas (OSAs).
– Mouse model revealed defects in generation of central respiratory rhythm, including reduced ventilatory response to CO_2_, and in the development of central noradrenergic (locus coeruleus) and serotonergic neurons.
– Respiratory instability (breathhold events) is also associated with deficient GABA-ergic transmission in the pontine Kolliker Fuse region (KF), the nucleus tractus solitarius (NTS), LC and ventrolateral medulla.
– Oxidative stress and lung inflammation are also hallmarks of the syndrome.

Congenital Central Hypoventilation Syndrome (CCHS)

– CCHS is a neurodevelopmental disorders due to heterozygous polyalanine expansion mutation (PARM) in the *PHOX2B* gene, a transcription factor involved in the development of the autonomic nervous system (ANS), including the neuronal structures that control breathing and integrate respiratory reflexes.
– Its estimated incidence is 1/148,000-1/200,000 live births (about 1,300 genetically confirmed cases worldwide).
– It is usually present at birth but late-onset (childhood and adulthood) cases have been diagnosed.
– Alveolar hypoventilation, due to reduced or absent ventilatory response to hypoxia and hypercapnia, is more severe during NREM sleep than during wakefulness.
– Respiratory phenotype in CCHS is mainly due to developmental defects in retrotrapezoid nucleus (RTN), a structure that integrates peripheral and central chemoreception, and that is missing in Phox2b/+7 alanine knock-in mouse model of CCHS. Defects in other areas, such as locus coeruleus (LC) have been reported in post-mortem brains of CCHS patients.
– Associated to CCHS, a spectrum of non-respiratory symptoms, including seizures, cardiac arrhythmias, congenital heart disease, ocular disorders, Hirschprung’s disease and neural crest tumors (neuroblastoma) reflects a more global ANS dysfunction.

A *PHOX2B* mutation would be always required for a diagnosis of CCHS; however, in individuals with CCHS or phenotypes similar to CCHS and negative for *PHOX2B* mutations, homozygous mutations in the *MYO1H* gene (Spielmann et al., 2017), and in *LBX1* gene (Hernandez-Miranda et al., 2018) have recently been found. In particular, LBX1 cooperates with PHOX2B in the development of the retrotrapezoid nucleus (RTN) (see below), and its frameshift mutation interferes with this cooperativity, probably by blocking the recruitment of co-activator and/or a possible interaction with PHOX2B, thus changing the way their target genes are regulated (see below).

The variable phenotypes reported in CCHS patients carrying the same mutation also suggest the involvement of modifier genes of expressivity (Di Lascio et al., 2018a; Bachetti and Ceccherini, 2020). Mutations in genes other than *PHOX2B*, involved in the differentiation of neural crest cells (*RET*, *GDNF*, *BDNF*, *GFRA1*, *PHOX2A*, *HASH-1*, *EDN1*, *EDN3*, *BMP2*) or in oncogenes (*BRAF*) (Bolk et al., 1996; Amiel et al., 1998; Weese-Mayer et al., 2002, 2003; Sasaki et al., 2003; Fernández et al., 2013; Al Dakhoul, 2017) have been found in some CCHS individuals. Remarkably, some of them are PHOX2B target genes (Flora et al., 2001; Bachetti et al., 2005), but the pathogenic role of these genetic variants is unclear (de Pontual et al., 2007).

### *PHOX2B* Mutations in CCHS

**FIGURE 1:**
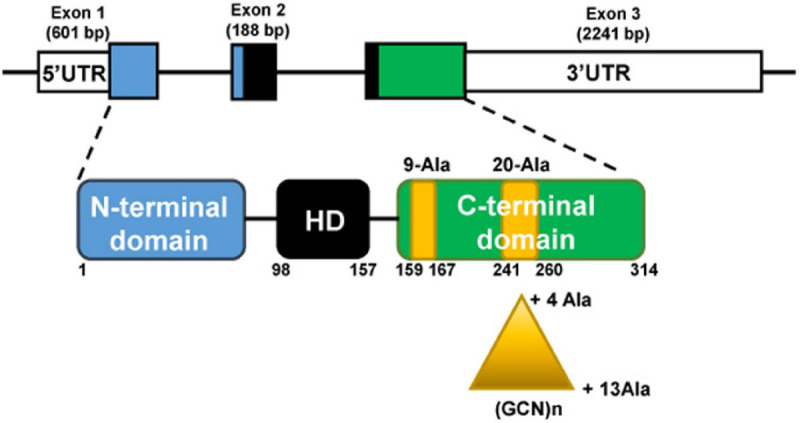
The *PHOX2B* gene **(top)** and protein **(bottom)**. The gene is composed of three exons; the protein presents an homeodomain (HD, black box) and 9 and 20 residues poly-alanine tracts (yellow boxes) in the C-terminus part. Triangle indicates triplet expansion of the 20 poly-alanine coding sequence, giving rise to the 4–13 polyalanine (polyAla) repeat expansion mutations (PARMs). The numbers indicate PHOX2B amino acid residues.

The *PHOX2B* gene is located at chromosome 4 (4p13) and is composed of three exons. PHOX2B protein is a transcription factor of 314 aminoacids, with a conserved 60-residues DNA binding domain (homeodomain) and two short and stable stretches of 9 and 20 alanine residues in the C-terminus (Figure 1).

The most frequent mutations found in CCHS patients are in-frame triplet duplications of different lengths within the sequence stretch coding for the 20 alanine tract. The polyalanine (polyAla) repeat expansion mutations (PARMs) lead to the addition of 4–13 alanine residues (normal genotype 20/20, CCHS genotypes 20/24–20/33), and short expansions of five, six, and seven alanines (genotypes 20/25, 20/26, 20/27) are the most frequent. Previous studies have tentatively described a genotype-phenotype correlation between the length of the expansion and the severity of the respiratory phenotype (Matera et al., 2004; Weese-Mayer et al., 2017; Di Lascio et al., 2018a,b; Bachetti and Ceccherini, 2020), but this relationship is variable (Trang et al., 2020). Homozygous individuals for the shortest polyalanine expansion (+ 4 alanines) have been described, whose phenotypes ranged from asymptomatic to severely affected (Trochet et al., 2008a; Repetto et al., 2009; Kwon et al., 2011; Chuen-im et al., 2014).

The minority of CCHS patients have heterozygous missense (MS), nonsense (NS) and frameshift (FS) mutations in exon 1, 2, or 3 [defined non-PARM mutations (NPARMs)]. The most frequent NPARM mutations are FS mutations that lead to a truncated or an elongated C-terminal region (reviewed in Weese-Mayer et al., 2017; Di Lascio et al., 2018b; Bachetti and Ceccherini, 2020), and can be associated with more severe syndromic forms, in which CCHS occurs together with HSCR and/or neuroblastoma (Matera et al., 2004; Berry-Kravis et al., 2006; Di Lascio et al., 2018b). Recently, it has been described a case of CCHS due to a combination of a PARM (+ 4 alanine) and a NPARM mutation, inherited from asymptomatic parents, each heterozygous for these *PHOX2B* variants (Sivan et al., 2019).

Polyalanine contractions have been reported in few CCHS or HSCR patients (Weese-Mayer et al., 2010; Di Zanni et al., 2017) and have also been found in a small percentage (1–1.5%) of the general population. Therefore, these variants are not considered diagnostic of CCHS, although they may have predisposing effects in HSCR and SIDS (Bachetti and Ceccherini, 2020, and references therein). A similar role has been hypothesized for the higher frequency of synonymous SNPs in *PHOX2B*, associated with OSAS (Lavezzi et al., 2013) and SIDS (Weese-Mayer et al., 2004), the reduced *PHOX2B* expression with protein dislocated in the cytoplasm in neurons from SIDS specimens (Lavezzi et al., 2012), and a complete *PHOX2B* gene deletion in an apparent life threatening event (ALTE) patient (Jennings et al., 2012).

## Modeling CCHS: *in vivo* and *in vitro* Approaches

Most of our knowledge about CCHS pathogenesis derives from the use of (i) neuroblastoma cell lines, (ii) animal models, with the possibility to derive tissue explants and organotypic cultures, and (iii) postmortem tissue analysis and biopsies. Although all of these approaches show some limitations, they have enabled significant strides toward understanding the pathological processes of the disease. Here we summarize the most important findings on PHOX2B physiological function and the molecular defects caused by its mutations, derived from *in vivo* and *in vitro* models of CCHS.

### *PHOX2B* in Neuronal Development and Cell Function

The *PHOX2B* gene is considered a “master selector gene” in the development of the ANS and the neural structures that are involved in breathing control and respiratory reflexes (Pattyn et al., 1999, 2000). In particular, central noradrenergic and hindbrain visceromotor neurons generation and maintenance, at least up to the late embryonic stages (Coppola et al., 2010; Fan et al., 2011), depends on PHOX2B, along with its paralog PHOX2A. In adult rats, *Phox2b* is expressed in several brainstem structures (Kang et al., 2007), although its exact role in adulthood is still to be defined. In particular, hypercapnia-sensitive neurons of the brainstem (Stornetta et al., 2006), including the neurons in the nucleus of the tractus solitarius (NTS) (Pattyn et al., 1997) and in the carotid bodies, express *Phox2b*. Moreover, *Phox2b*^+^/*VGlut2^+^/Nmb^+^* glutamatergic neurons located on the ventral surface of the medulla oblongata (Stornetta et al., 2006; Shi et al., 2017), below the facial motor nucleus, form the retrotrapezoid nucleus (RTN). This structure is involved in integrating peripheral and central chemoception (Dauger et al., 2003; Mulkey et al., 2004; Takakura et al., 2006; Guyenet and Bayliss, 2015), and mediating most of the ventilatory reflex to hypercapnia, particularly during NREM sleep (Guyenet et al., 2016, 2019). The dependence on *Phox2b* expression of all these structures comes from the observation that they are missing in *Phox2b* knock-out mice (Pattyn et al., 1999; Dauger et al., 2003).

PHOX2B, by means of its homeodomain region and the C-terminal domain, forms homo- and heterodimers with other homeoproteins, including its paralog PHOX2A (Adachi et al., 2000; Di Lascio et al., 2016a). The C-terminal domain containing the two polyalanine stretches and in which the PARMs and the majority of NPARMs mutations are located is also involved in DNA binding affinity and solubility of the protein (Di Lascio et al., 2016a).

So far, several hypotheses have been proposed to find a role for polyalanine tracts (single amino acid repeats), among which flexible spacer linking functional protein domains, drivers of protein conformation, protein-protein interactions, and DNA-binding, thus modulating transcription factor activity (Di Lascio et al., 2018a). Indeed, it has been demonstrated that the presence of the 20-alanine stretch is important for PHOX2B transcriptional activity (Radó-Trilla et al., 2015), but it is not required for protein localization and its ability to form dimers (Di Lascio et al., 2013, 2016a).

### Developmental Defects in Mouse Genetic Models and CCHS Patients

To investigate the developmental defects occurring in CCHS patients, several mouse models have been generated (reviewed in Amiel et al., 2009; Moreira et al., 2016; Di Lascio et al., 2018a). Constitutive and conditional knock-in (KI) models, carrying both PARM and NPARM *PHOX2B* mutations, show impaired CO_2_ chemosensitivity and the selective deletion of the RTN as a common trait, thus indicating it as the main cause of the respiratory phenotype. However, it is unlikely that the respiratory deficits observed in some patients during wakefulness could be explained by RTN absence or defects. Indeed, it has been hypothesized (Tremoureux et al., 2014) that, in wakefulness, activation of motor areas (i.e., cortex) may compensate for the defect in brainstem centers controlling autonomic breathing. In CCHS patients it has been reported the existence of a “resource competition” (Sharman et al., 2014) between control of breathing by cortical areas and other cortical functions, that require mental concentration (such as watching television, video gaming, and studying) resulting in increased hypoventilation in CCHS patients, and indeed mechanical ventilation during wakefulness ameliorates cognitive performances. Until now, the existence and location of RTN have been only tentatively defined in humans (Rudzinski and Kapur, 2010; Lavezzi et al., 2012) and the recent analyses on post-mortem brains of CCHS patients did not confirm defects in any RTN-like structure (Nobuta et al., 2015). Conditional KI mice, carrying a NPARM *PHOX2B* mutation, shed light in filling the gap between mice and human manifestation of the disease, indicating that the chemosensory control of breathing not only depends on RTN function, but also on noradrenergic neurons of *locus coeruleus* (LC), a neuronal structure that in +7 Alanine KI animals apparently developed normally (Dubreuil et al., 2008; Nobuta et al., 2015; Moreira et al., 2016). In NPARM KI mice, the mutation inhibits LC precursors differentiation, in addition to the loss of the RTN, facial nerve nucleus and intestinal aganglionosis. *PHOX2B* mutations therefore prevent neuronal development at different stages with variable outcomes (from complete loss to incomplete differentiation), and it is likely that both RTN loss and LC impairment contribute to the respiratory deficits of CCHS patients. Consistently, LC defects have recently been confirmed in two CCHS cases with different *PHOX2B* mutations (one PARM and the same NPARM of conditional KI animals) (Nobuta et al., 2015).

The existence of a LC-preBötzinger complex circuit controlling breathing has recently been demonstrated by Liu et al. (2020) by showing that selective stimulation of Phox2b^+^ LC neurons enhanced basal ventilation in conscious mice, that Phox2b^+^ LC neurons lesions by genetic manipulation reduced hypercapnic ventilatory responses, and that LC neurons are physically connected, by axons projection, to the complex. LC defects are also an hallmark in models of Rett syndrome (Roux et al., 2010), that share with CCHS respiratory defects, although more severe during wakefulness than during sleep (Weese-Mayer et al., 2008; Ramirez et al., 2020).

Recently, it has been shown that mutant Phox2b may induce secondary/non-cell autonomous defects of key brainstem respiratory neurons (Alzate-Correa et al., 2020). Indeed, the expression of NPARM *PHOX2B* mutation in Phox2b^+^ non-respiratory progenitor cells such as visceral motor neuron progenitors, induced a severe neonatal apnea along with a significant loss of neurons directly deriving from that specific progenitor domain but also from respiratory neural structures, such as RTN and preBötC, embriologically unrelated to that progenitor domain.

CCHS patients also have cardiovascular, gastrointestinal and ocular deficits that indicate additional brainstem defects, consistent with the recent brain imaging studies (Harper et al., 2015).

Recently, the role of astrocytes in the chemosensitive response (paracrine hypothesis) has been postulated (Guyenet et al., 2019, and references therein). In particular, it has been reported that PHOX2B-derived astrocytes play a role in chemosensory control of breathing (Czeisler et al., 2019), especially in the adult, by maintaining a functional O_2_ chemosensitive response, adequate sleep homeostasis and by ensuring synaptic integrity of neurons in RTN. It is then plausible to hypothesize that *PHOX2B* mutations may have consequences also in the development of this group of astrocytes. This finding adds a level of complexity in the understanding of neuronal control of breathing, but unraveling the molecular mechanism of such a control may represent a new challenge into the comprehension of CCHS pathogenesis.

### Mutant PHOX2B Proteins Show Altered Properties and Functional Activities

**FIGURE 2:**
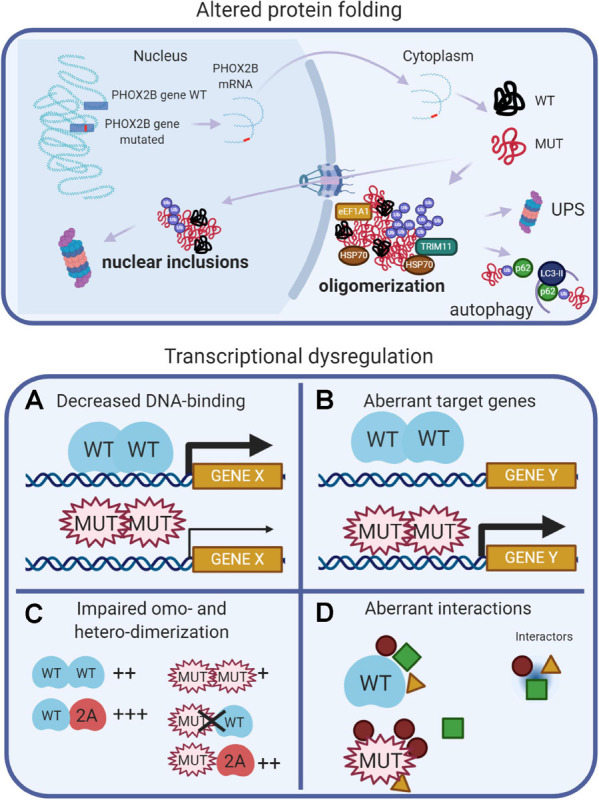
(Created with BioRender.com). Molecular basis of CCHS. The upper panel shows that, in CCHS, mutations affect one copy of the *PHOX2B* gene. Transcription and translation give rise to two proteins, wild-type and mutated. Misfolded mutant proteins form aggregates in the cytoplasm, also sequestering wild-type proteins, and may be bound by molecular chaperones (heat shock proteins, HSPs) in an attempt to refold them. Alternatively, unsuccessful folding can facilitate their ubiquitination (Ub), a process involving ubiquitin ligases such as TRIM11, and recognition by the proteolytic machinery (UPS, Ubiquitin Proteasome System). Autophagy can participate in the removal of PHOX2B protein aggregates. A fraction of the mutant proteins can enter the nucleus, aggregate and form nuclear inclusions or induce transcriptional dysregulation (lower panels) by: **(A)** decreasing DNA binding, limiting PHOX2B transcriptional activity; **(B)** inducing aberrant genes expression; **(C)** decreasing the formation of homodimers and the interaction with the paralog PHOX2A; **(D)** aberrant interactions with transcriptional cofactors; some of these might be inappropriately enhanced, whereas others might be lost or unchanged.

Data from experimental models of CCHS suggest that different pathogenetic mechanisms (loss-of-function, dominant-negative, and toxic effects of the mutant proteins) may contribute to explain the entire spectrum of CCHS. These mechanisms are recapitulated in Figure 2, and extensively reviewed (Di Lascio et al., 2018a, and references therein). Protein misfolding and transcriptional dysregulation are among the most studied.

Protein misfolding has been extensively studied *in vitro:* both PARM and NPARM mutations form oligomers (Di Lascio et al., 2018a) and recently, it has also been reported a tendency of the + 7 alanine variant to form fibrils (Pirone et al., 2019). In cell models, only longer polyalanine expansions caused cytoplasmic aggregation (Di Lascio et al., 2018a, and references therein) and it has been proposed that the polyalanine tract may play a role in the cytoplasmic mislocalization of transcription factors containing poly(A)-tracts, acting as a nuclear export signal (NES) (Li et al., 2017), by means of interaction with the elongation factor 1A1 (eEF1A1). The reduction of *eEF1A1* restores the nuclear localization of poly(A)-containing transcription factors and their transcriptional activities. Moreover, a fraction of PHOX2B wild-type protein is sequestered by the mutant protein in the cytosol, thus having a dominant negative effect on the localization and activity of the normal protein (Parodi et al., 2012; Di Lascio et al., 2013).

Conversely, frameshift mutations retain nuclear localization, but formed inclusions and accumulate in the nucleoli (Di Lascio et al., 2018b; Ye et al., 2019). It is worth noting that the *in vivo* aggregation of PHOX2B protein has not been reported so far, and so the role of aggregates and their toxic effects in CCHS is unclear. In favor of the aggregation theory, there is the finding that anesthetic agents can promote LO-CCHS, by inducing aggregation and mislocalization of PHOX2B variants via the endoplasmic reticulum (ER) unfolded protein response (UPR) (Basu et al., 2017; Coghlan et al., 2018).

Studies of cell models of CCHS indicate that, along with protein misfolding, transcriptional dysregulation may be another important pathogenetic mechanism (Figure 2). PARMs and NPARMs both show decreased DNA binding capability and transcriptional activity (Figure 2A), and mutant proteins reduce the activation of some PHOX2B target genes (Nagashimada et al., 2012; Di Lascio et al., 2013, 2018b), as reviewed in Di Lascio et al. (2018a). Moreover, PHOX2B also transactivates its own promoter (Cargnin et al., 2005), and the mutations may also interfere with this auto-regulation mechanism thus contributing to a reduction of the amount of normal PHOX2B protein.

It has also been reported that frameshift mutations aberrantly regulate some target genes (Figure 2B), such as *SOX10* and the glial acidic fibrillary protein (*GFAP*) (Nagashimada et al., 2012; Di Lascio et al., 2018b).

PHOX2B forms homodimers and heterodimers with PHOX2A (Adachi et al., 2000; Di Lascio et al., 2016a; Moreira et al., 2016) and dimerization is crucial for DNA-binding and transcriptional activity (Figure 2C). Alanine expansions progressively reduce the formation of dimers and PARM mutants interact weakly with normal protein (Di Lascio et al., 2016a); some of the observed dominant-negative effects are likely to be caused by the aberrant interactions with transcriptional co-activators or co-repressors (Figure 2D; Wu et al., 2009; Reiff et al., 2010).

It is interesting to note that PHOX2B mutants partially form heterodimers with PHOX2A retaining partial transcriptional activity, and they do not interfere with the localization and transcriptional activity of PHOX2A (Di Lascio et al., 2016a). This mechanism can also explain the CCHS cases in which a mutated partner of PHOX2B (namely LBX1) can interfere with the correct recruitment of co-activator necessary for the correct expression of PHOX2B target genes (Hernandez-Miranda et al., 2018). In brief, *PHOX2B* mutations cause a combination of loss-of-function, dominant-negative, and, gain-of-function effects and the entity of the transcriptional dysregulation is gene specific. Therefore, it is likely that the number and importance of the dysregulated target genes depends on the different types of mutations and this eventually determines the severity of the disease.

**FIGURE 3:**
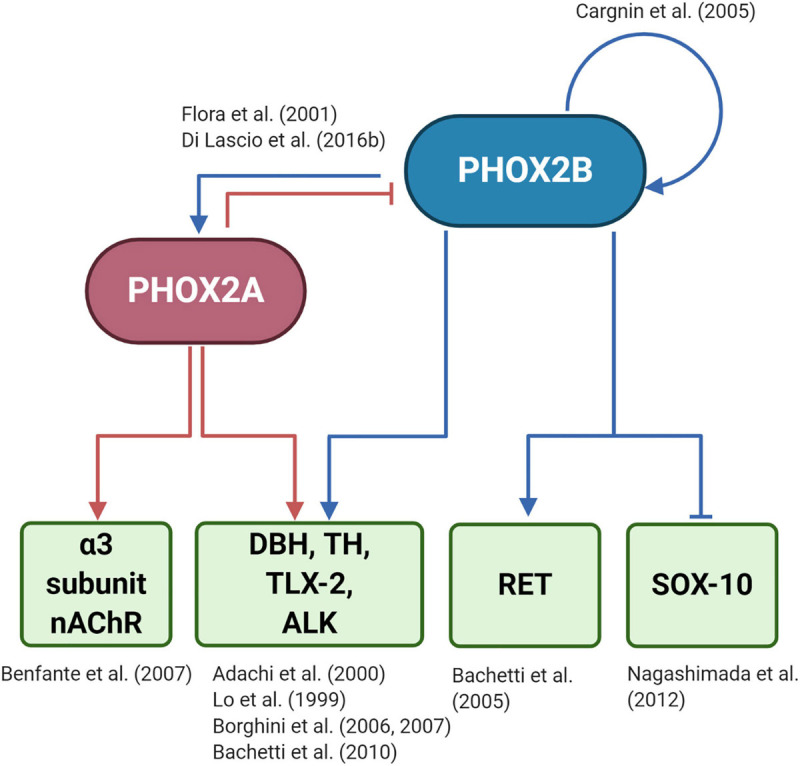
(Created with BioRender.com) Target genes regulated by PHOX2B and PHOX2A. The diagram shows that PHOX2B regulates itself (Cargnin et al., 2005). Expression of the paralog gene *PHOX2A*, which is important for the expression of pan-autonomic genes as the α3 subunit of the ganglionic type nAChR (Benfante et al., 2007), depends on PHOX2B (Flora et al., 2001). PHOX2A, in turn, negatively regulates *PHOX2B* (Di Lascio et al., 2016b). Tyrosine hydroxylase (*TH*) and Dopamine-Beta-Hydroxylase (*DBH)*, the rate-limiting enzymes of catecholamine biosynthesis (Lo et al., 1999; Adachi et al., 2000), TLX-2 (Borghini et al., 2006, 2007), a transcription factor involved in the development of enteric nervous sytem, and ALK (Bachetti et al., 2010), a tyrosine kinase whose mutations are associated with neuroblastoma, are regulated by both PHOX2 proteins. *RET* that is the most important gene driving the complex inheritance of Hirschsprung’s disease (HSCR), is indirectly regulated by PHOX2B (Bachetti et al., 2005), as *RET* promoter does not contain a PHOX2B responsive element. The inhibition of *SOX10* by PHOX2B is critical for the differentiation of bipotential neural crest derived progenitors toward neuronal lineage (Nagashimada et al., 2012).

However, only a few PHOX2B regulated genes have been identified so far (Figure 3): tyrosine hydroxylase (*TH*) and dopamine-beta-hydroxylase (*DBH*), (Lo et al., 1999; Adachi et al., 2000); *PHOX2A* (Flora et al., 2001); *TLX2* (Borghini et al., 2006; Borghini et al., 2007); *RET* (Bachetti et al., 2005); *SOX10* (Nagashimada et al., 2012); *ALK* (Bachetti et al., 2010), and *PHOX2B* itself, because its expression depends on an auto-regulatory mechanism (Cargnin et al., 2005). To better understand the entire spectrum of CCHS, the discovery of all PHOX2B target genes is mandatory.

## Therapeutic Research Based on Advances in Our Understanding of CCHS

The diagnosis and treatment of CCHS patients have made great progress after the discovery of *PHOX2B* as the disease causing gene; on the other hand its role as a regulator of the development of ANS arised some concerns regarding the possibility to explore a pharmacological intervention in CCHS. The failure of the development of the neuronal structure driving the chemosensitive response to hypoxia and hypercapnia in CCHS transgenic mice models and possibly in CCHS patients (i.e., the RTN), has been considered an insurmountable obstacle toward a therapeutic intervention, supported by the findings that damages to these and other neuronal structures can progressively occur (Harper et al., 2015), probably due to postnatal hypoxic and hypercapnic episodes or to the persistence of the detrimental effects of *PHOX2B* mutations in vulnerable cell populations.

A residual cardiorespiratory response has been reported in some younger CCHS patients, an observation that can be explained by the fact that the loss of RTN described in a mouse models with a + 7 alanine mutation is not complete, but limited to 70% of the cells (Dubreuil et al., 2008). Moreover, it cannot be excluded that other chemosensitive areas are activated to compensate for the loss of RTN.

These findings pave the way for the development of therapeutic strategies even after birth, as supported by studies of other neurodevelopmental disorders (Castrén et al., 2012; Matagne et al., 2017).

Another unexplored aspect of CCHS is how it can progress with age, in particular with respect to autonomic or cognitive functions; we can hypothesize that tuning the expression of *PHOX2B* and its target genes, as done by using retinoic acid (Di Lascio et al., 2016b), may divert the natural history of the disease. In this sense, a very recent study showed that forcing Phox2a/2b expression, in the LC of aged rats results in increased neurogenesis in the hippocampal dentate gyrus, increased norepinephrine levels in the striatum, and improved cognitive behavior, thus suggesting that Phox2a/2b play an important role in recovering noradrenergic and dopaminergic neurons function in aged animals (Fan et al., 2020).

In these perspectives, we report therapeutic approaches that have been exploited, *in vitro*, in order to reduce the damage caused by aberrant function of mutant PHOX2B. Furthermore, the serendipitous observation in two CCHS patients that chemosensitivity can be partially restored upon treatment with the progestin desogestrel has strong proof of concept value for a therapeutic approach in CCHS, at least in terms of relieving respiratory symptoms.

### *In vitro* Studies

Based on *in vitro* studies on the mechanisms of CCHS pathogenesis summarized in Figure 2, the main goal of current therapeutic research, at the molecular level, is to hamper the toxic effects of mutant PHOX2B, focussing on molecules that can re-establish PARM-bearing mutant proteins correct localization and function, not only limited to CCHS (Di Zanni et al., 2011). As polyalanine expansions induce the formation of protein aggregates (Di Zanni et al., 2011; Pirone et al., 2019), possible target for pharmacological treatments are the pathways involved in the control of the protein quality (i.e., proteasome, autophagy and heat-shock pathways) with the aim to induce their activity to remove mutated proteins (Bachetti et al., 2007; Di Zanni et al., 2011, 2012; Parodi et al., 2012), as shown for geldanamycin and 17-AAG. These molecules, by recovering folding and correct localization to the nucleus, limited the dominant-negative effect of mutant PHOX2B protein on wild-type protein (Di Lascio et al., 2013). An alternative degradative pathway to reduce PHOX2B protein aggregates is mediated by autophagy (Bachetti et al., 2007; Di Zanni et al., 2012). Antioxidant therapies may also be beneficial in relation to the increase in ROS found in CCHS patients (Degl’Innocenti et al., 2018); curcumin, a major component of turmeric (*Curcuma longa)*, promoted PHOX2B refolding (Di Zanni et al., 2012) without inducing the expression of molecular chaperones. It can be reasonable to hypothesize that different pathways (reviewed in Carvalho et al., 2016), based on its potent anti-inflammatory and anti-oxidant activities, may be involved.

The recent findings of the involvement of eEF1A1 in the mislocalization of polyalanine expanded protein into the cytoplasm, make this factor a new druggable target for poly(A) diseases (Li et al., 2017).

Another level of therapeutic intervention relies on the fact that many genes are regulated by PHOX2B, and those genes might be potential target for treating the disease. Eventually, the disease has to be considered the result of the aberrant expression (absent, down- or up-regulation expression) of PHOX2B target genes.

Unfortunately, very few genes are known to be PHOX2B targets (Figure 3), despite the fact that filling this gap is really urgent.

### *In vivo* Studies

#### The Use of Progestins in CCHS: The Case of Desogestrel

In 2010, Straus et al. reported the fortuitous observation that two female patients (respectively, with 20/25 and 20/26 genotypes) underwent a partial recovery of chemosensitivity and increased ventilation when using the progestin drug desogestrel (13-ethyl gonanes family) for contraceptive purposes (Straus et al., 2010), although the magnitude of improvement was not sufficient to replace assisted ventilation (Straus and Similowski, 2011). This effect was not replicated on another CCHS patient (Li et al., 2013).

**FIGURE 4:**
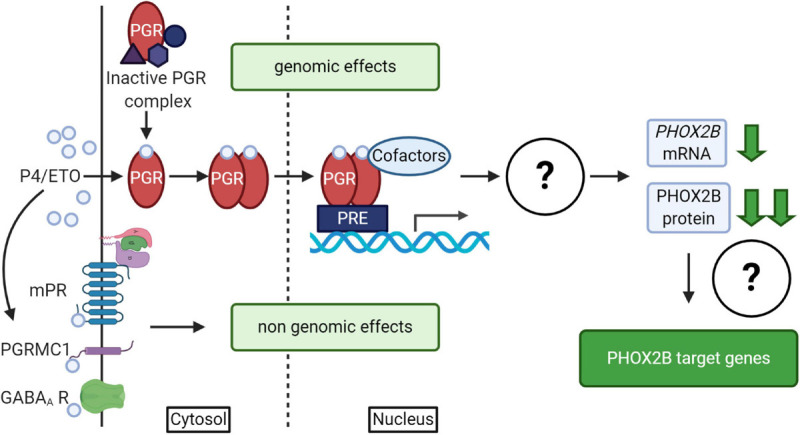
(Created with BioRender.com). Schematic diagram of progesterone (P4)/progestins signaling pathways. Genomic effects of P4/ETO are mediated by nuclear progesterone receptor (PGR) which is inactive in the cytosol until progesterone binding. After P4 binding, it form dimers, translocates to the nucleus and dimers bind to Progesterone Responsive Element (PRE) within gene promoters and regulate the expression of target genes. The scheme also shows that the activation of PGR receptor by ETO is able to induce *PHOX2B* down-regulation by unknown mechanisms (Cardani et al., 2018), with possible effects on the expression of some PHOX2B target genes. Non-genomic effects of P4/ETO are mediated by the binding of P4 to cognate membrane receptors (mPR and PGRMC1) or to those belonging to other neurotransmitters (such as GABA_*A*_ receptors).

It is well known that progesterone, besides the reproductive and neuroprotective effect (Schumacher et al., 2014), powerfully stimulates respiration; indeed it has been used for the treatment of both adult apnea and of apneic pre-term neonates (Bairam et al., 2019, and references therein). Progesterone induces both genomic and non-genomic effects (Singh et al., 2013; Stanczyk et al., 2013; Schumacher et al., 2014) by the activation of nuclear (PGR) or membrane (mPR) receptors (Brinton et al., 2008; Figure 4); it has been shown that both receptors have a role in the modulation of chemoreflex response and respiratory control particularly during sleep (Bairam et al., 2019, and references therein). The exact molecular mechanism underlying this pharmacological effect is unknown; recently, *in vitro* studies showed that PHOX2B and desogestrel are molecularly linked demonstrating that the biologically active metabolite of desogestrel, 3-Ketodesogestrel (3-KDG; etonogestrel, ETO), modulates both wild type and mutant *PHOX2B* and the expression of its target genes via progesterone nuclear receptor PR-B (PGR) (Cardani et al., 2018). Remarkably, the expression of both wild-type and mutated PHOX2B is negatively regulated by 3-KDG (Figure 4). The exact mechanism and the molecular factor(s) involved are still unknown; however, it has been hypothesized that post-transcriptional mechanisms, including translation inhibition, could mediate 3-KDG effect on PHOX2B. As this process does not require the synthesis of new molecules, post-translational modifications (i.e., phosphorylation/de-phosphorylation), may be involved in mediating the effect of 3-KDG. In agreement with this hypothesis, it is known that monoubiquitylation of Phox2b/Phox2a by Rnf220/Zc4h2 complex is required for normal central noradrenergic neuron differentiation (Song et al., 2020).

PHOX2B downregulation by ETO does not apparently support the results reported by the clinical observation (Straus et al., 2010). In fact, 3-KDG decreases *PHOX2B* expression and haploinsufficiency is one of the mechanisms proposed in the insurgence of CCHS. However, as we described, respiratory defects may also depend on the gained toxic functions by mutant proteins (Di Lascio et al., 2018a), leading us to hypothesize that the positive clinical effect may be explained by the beneficial decreased level of the mutant PHOX2B, capable of counteracting the potential pathogenic effect of insufficient *PHOX2B* expression.

Data reported in Cardani et al. (2018) also showed that a slight PHOX2B decrease does not cause a down-regulation of all PHOX2B target genes, and in cell lines expressing *PHOX2A* and low levels of *PHOX2B*, progesterone may have the paradoxical effect of inducing tyrosine hydroxylase (Jensik and Arbogast, 2011), a well-known PHOX2B target gene. These findings suggest that *PHOX2B* expression level and the targeted cell-type may determine the positive or negative progesterone-mediated effects.

**FIGURE 5:**
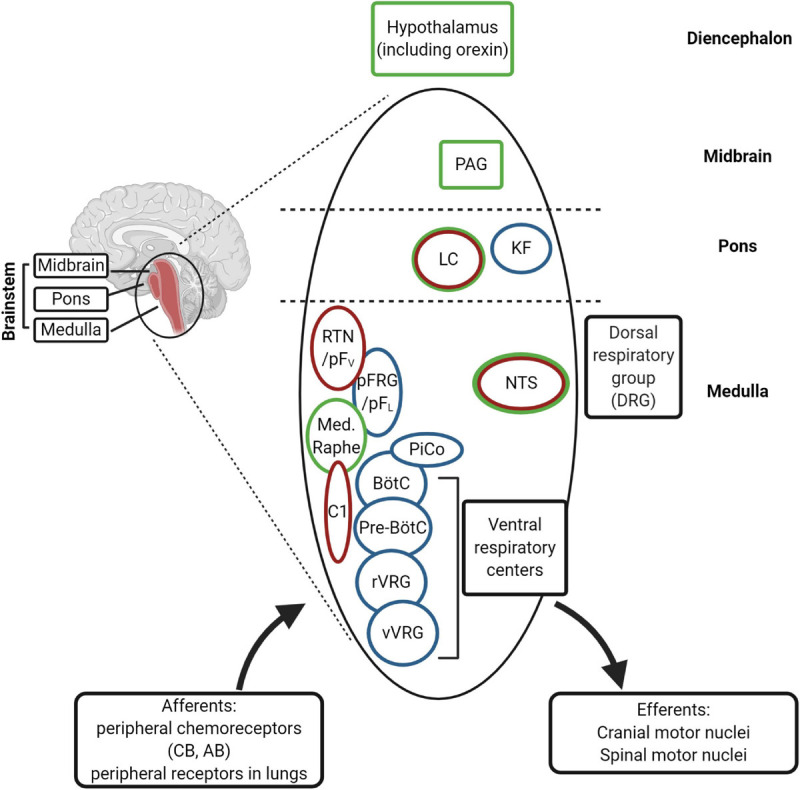
(Created with BioRender.com). Schematic diagram of the main respiratory centers in the brainstem. The rhythm generators are the pre-Bötzinger complex (pre-BötC) (inspiratory), the postinspiratory complex (PiCo), and lateral pFRG/RTN (expiratory). The RTN (retrotrapezoid nucleus) plays an important role in central chemosensitivity and receives inputs from NTS (nucleus of the solitary tract). The neural network integrates afferent information from the periphery with central chemoreception signals and activates efferent motor neurons [through rVRG (rostral ventral respiratory group) and cVRG (caudal ventral respiratory group), among others]. The rhythm generators receive inputs from serotonergic (medullary raphe), adrenergic (C1 neurons) and cholinergic neurons (Kolliker-Fuse nucleus, KF) of the ponto-medullary regions for modulation of the respiration in the different behavioral states, including sleep and wakefulness. The medullary respiratory centers are also connected to higher centers in the pons (LC, locus coeruleus), in the midbrain (PAG, periaqueductal gray) and diencephalon (hypothalamus). The nuclei expressing *Phox2b* are indicated in red and those expressing PGR and/or progesterone-sensitive in green. CB, carotid body; AB, aortic bodies.

Among neuronal structures well known targets for progesterone are medulla oblongata, midbrain and diencephalon (Figure 5). In young mice, Pgr is expressed in different CNS structures (Quadros et al., 2007, 2008), including those areas that control breathing (Benarroch, 2018), but only in a few of those Pgr turned out to be co-expressed with PHOX2 proteins (Table 1; Kang et al., 2007; Quadros et al., 2008; Card et al., 2010).

**TABLE 1:** Brain distribution of Phox2a, Phox2b, and progesterone receptor (Pgr) immunoreactivity in adult rats.

**Adult rat (brain area)**	**Phox2a Card et al., 2010**	**Phox2b Kang et al., 2007**	**Pgr Quadros et al., 2007, 2008**
**Brainstem**			
**Medulla**			
Nucleus of the solitary tract (NTS)	**+**	**+**	**+**
Area postrema (AP)	**+**	**+**	−
Dorsal motor vagal nucleus (DMV)	**±**	**+**	−
Inferior salivatory nucleus	**+**	−	−
Subjacent to the fourth ventricle	**+**	**+**	**±** (vestibular nucleus)
**Ventrolateral brainstem (Medulla and pons)**			
Rostroventral medulla (C1 neurons)	**+**	**+**	−
Retrotrapezoid nucleus (RTN)	**+**	**+**	−
Superior salivatory nucleus	**+**	**+**	−
Nucleus ambiguus	−	**+**	−
**Pons**			
Locus coeruleus (LC)	**+**	− *	−
Parabrachial nucleus, lateral	−	−(rare)	**+**
Parabrachial nucleus, medial	**+**	−(rare)	**+**
Raphe pontis nucleus	−	−	**+**
Pedunculopontine tegmental nucleus	−	−	**+**
**Midbrain**			
Periaqueductal gray	**+**	−	**+** (not ventral)
A7 cell group	**+**	−	−
Substantia nigra, compact	−	−	**+**
Ventral tegmental Area	−	−	**+**
Retrorubral field	−	−	**+**
Inferior colliculus	−	−	**+**
Anterior pretectal nucleus	−	−	**+**
**Diencephalon**			
Dorsomedial hypothalamic nucleus (DMH) in caudal hypothalamus	**+**	−	**+**
Ventrolateral preoptic nucleus (VLPO) in rostral hypothalamus	**+**	−	**+** (preoptic area)
Retrochiasmatic area and periventricular nucleus near suprachiasmatic nuclei	**+** (scattered neurons)	−	−

+ and − indicates presence or absence, respectively. *Phox2b mRNA has been detected in the locus coeruleus of adult rats by in situ hybridization (Fan et al., 2011).

Interestingly, the NTS, located in the dorsal medulla and known to be indispensable for the integration of sympathetic and respiratory responses to hypoxia and hypercapnia (Zoccal et al., 2014; Fu et al., 2019), is the only structure which co-expresses Phox2a, Phox2b, and Pgr (Table 1), and it has been reported that NTS neurons respond to progesterone and 3-KDG administration (Pascual et al., 2002; Loiseau et al., 2014; Joubert et al., 2016).

The reticular formation nuclei, such as the Periaqueductal Gray (Subramanian et al., 2008; Lopes et al., 2012), and the Parabrachial nucleus (Zuperku et al., 2019) are particularly interesting, because they co-express Pgr and Phox2a and it is known to play a role in breathing modulation (Card et al., 2010). Another structure that shows a Phox2a/Pgr restricted expression is hypothalamus, in particular the Dorsomedial hypothalamic nucleus (DMH), in caudal hypothalamus, and the Ventrolateral preoptic nucleus (VLPO), in rostral hypothalamus, both found to be important in the control of behavioral state (Saper et al., 2005). Moreover, VLPO controls the wake-sleep cycle regulation (Saper et al., 2005), whereas chemical stimulation of DMH has been associated to increased phrenic nerve stimulation (Tanaka and McAllen, 2008). The lateral area of hypothalamus contains CO_2_/H^+^ chemosensitive orexin neurons (Wang et al., 2018), and it has been reported that they contribute to the hypercapnic ventilatory response (Loiseau et al., 2019, and references therein), in a state-dependent manner (Rodrigues et al., 2019). These neurons have recently been involved in the desogestrel effect (Loiseau et al., 2014, 2018, 2019). In particular, data from Bodineau’s group (Loiseau et al., 2014; Joubert et al., 2016) suggest that two distinct pathways, medullary and supra-medullary, are involved in the ETO ventilatory effects, excluding the involvement of RTN in the progestin effect.

By working on *ex vivo* mouse medullary-spinal cord preparations or *in vivo* newborn mice from both genders exposed to ETO, Loiseau et al. (2019) identified in orexin neurons the target of ETO-induced facilitation on respiratory frequency increase, following metabolic acidosis, and suggested that this was due to a non-genomic effect of desogestrel (Singh et al., 2013). The presence of PR receptors (nuclear and/or membrane) in orexin neurons is still unknown; however, the role of membrane progesterone receptor (mPR) could be excluded because progestins have little if any binding affinity for this membrane receptor (Giatti et al., 2016). Besides this, it has been hypothesized that ETO effect could be mediated by the modulation of other neurotransmitter systems. Indeed, progesterone and its metabolites act as allosteric modulator of GABA_*A*_ receptor, potentiating GABA-induced chloride conductance (Figure 4), and of other Cys-loop family receptors (i.e., AChR, glycine, and 5-HT3), but also NMDA (NMDAR) and kainate receptors. Steroids, by binding NMDAR, potentiate the NMDA induced increase in respiratory frequency. However, whether synthetic progestin metabolites modulate GABA_*A*_ receptor is still a matter of debate (Giatti et al., 2016; Joubert et al., 2016; Loiseau et al., 2018).

In conclusion, Loiseau et al. (2019) suggested that ETO induces the CO_2_/H^+^ chemosensitivity improvement in some CCHS patients by stimulating, or potentiating still functioning CO_2_/H^+^ chemosensitive central structures. However, potential limitations of this work are that the study has been conducted in newborn wild type animals, whereas the two CCHS patients in Straus’ study are adult, and the neuronal structures might be immature compared to adult humans. Indeed, in CCHS patients ETO potentiates also basal ventilation (Joubert et al., 2016; Loiseau et al., 2018) that may require the functioning of serotoninergic neurons within the medullary raphe nuclei, and modulation of GABA_*A*__–_ and NMDA-mediated ventilatory regulations (Loiseau et al., 2018). Furthermore, the use of wild type animals has not allowed to explore whether orexin neurons stimulation still contributes to respiratory improvement in the presence of *PHOX2B* mutations.

The range of effective ETO concentration was very narrow and according to authors (Loiseau et al., 2019) the dose used in another unresponsive CCHS patient was inappropriate, thus explaining the observed unresponsiveness to desogestrel (Li et al., 2013). Altered ETO metabolism and/or impaired permeability to steroids of the blood-brain-barrier may be the cause (Stanczyk et al., 2013) and suggests that the effective desogestrel dose has to be personalized.

However, the reported improvement of respiratory parameters in two CCHS patients by ETO may also be explained by desogestrel activation of other areas of rodent brain (Loiseau et al., 2014; Joubert et al., 2016), including some expressing *Phox2b* in adulthood, as the C1 neurons (Stornetta et al., 2006), and catecholaminergic neurons in the ventrolateral medullary reticular nucleus (Joubert et al., 2016).

These data, along with *in vitro* and *in vivo* studies previously described, indicate that 3-KDG may have a more complex effect on general ventilation in CCHS patients acting on different respiratory networks.

#### Other Pharmacological Targets

Recently, a child with CCHS due to heterozygous missense variant in exon 1, c.95A > T, showed an improvement in his daytime apneic episodes following treatment with carbamazepine (Schirwani et al., 2017), with no effects on sleep-related hypoventilation. Carbamazepine is mainly used as antiepileptic drug, because of its ability to reduce the iperexcitability of neurons by blocking voltage-gated sodium channels. It is also used as a mood stabilizer, especially to control maniac states, by exploiting its possible effects on noradrenaline in the brain. To explain the clinical improvement observed in the patient it has been hypothesized that carbamazepine may act by antagonizing A1 and A2 adenosine receptor, or by blocking noradrenaline receptors. Consistently, adenosine acts as a respiratory depressant, and adenosine antagonists are efficient respiratory stimulants, already used to treat neonatal apnea (Hascoet et al., 2000).

Another important class of possible drug targets is represented by ion channels. Neuronal cell excitability relies on their proper function, and in particular it has been reported that K^+^ channels play a role in neuronal development and their activity modulates respiration *in vivo* (Malin and Nerbonne, 2002; Zavala-Tecuapetla et al., 2008; Lazarenko et al., 2010; Trapp et al., 2011; Torrecilla et al., 2013; Hawkins et al., 2015; Sobrinho et al., 2016).

Works by Bayliss’ lab demonstrated that the intrinsic chemosensitivity of RTN neurons is due to two independent molecular pH sensors, namely TASK-2 (Wang et al., 2013; Bayliss et al., 2015), an alkaline-sensitive K^+^-channel of the two-pore domain (K2P) family, and GPR4 (Kumar et al., 2015), a proton activated receptor. Genetic deletion of both genes recapitulates the cardinal features of CCHS patients. On the other hand, these mice showed a residual chemosensitivity of RTN neurons and of the respiratory network, thus suggesting the activation of alternative/compensatory cellular and molecular mechanisms (Guyenet et al., 2016).

Recently (Mulkey et al., 2015), the KCNQ channels family has been reported to determine the chemoreceptor function of RTN, whereas the overexpression of the *KCNN3* gene induces abnormal respiratory responses to hypoxia (Mahida et al., 2014) and increased risk of sudden cardiac death (Bond et al., 2000; Mahida et al., 2014), thus indicating a possible use of drugs targeting them for ameliorating respiratory dysfunctions.

Several studies demonstrated that hypercapnia stimulates LC and C1 group neurons and has an excitatory effect on brainstem respiratory neurons (Viemari, 2008; Gargaglioni et al., 2010; Magalhães et al., 2018). These neurons express several chemosensitive K^+^ channels (Gargaglioni et al., 2010, and references therein), among which the pH-sensitive Kir channels (D’Adamo et al., 2011), the leak-conductance two-pore domain TASK-1 channels (Bayliss et al., 2001), the large conductance Ca^2+^-activated K^+^ (BK) channels (Imber et al., 2018), the Kv channels and the acid-sensing ion channel (ASICs). The presence of multiple pH-sensitive ion channels, which may have different sensitivity to pH variation, can result in a fine modulation of neuronal response to hypercapnia, by means of a mechanism mainly based on inhibition of K^+^ channels. The transient receptor potential (TRP) channels have also been implicated in the excitability of LC neurons in the presence of 8% CO_2_ (Cui et al., 2011). They are Ca^+2^ channels able to depolarize CO_2_-sensitive neurons and their dysregulation could result in respiratory disorders.

The importance of ion channels as druggable targets in CCHS is supported by the commercial availability of drugs that potentiate or inhibit their function, already used in different clinical settings.

## Conclusion and Future Perspectives

The knowledge acquired so far on *PHOX2B* and its mutation, and its role in the etiogenesis of CCHS have strongly contributed to the improvement of the diagnosis and treatment of the patient, including the attempt to predict the severity of the disease by genotype-phenotype correlation analyses. The study of the impact of *PHOX2B* mutations on its functional activity has shed light on the identification of possible pharmacological targets to limit and/or counteract the toxic effect of mutant PHOX2B in order to improve respiratory symptoms. Among these, its target genes certainly represent the greatest challenge, as transcriptional dysregulation is among one of the pathogenetic mechanism involved.

The serendipitous observation about progestin improvement of respiratory parameters in two CCHS patients, and the evidence that younger patients show a residual cardiorespiratory response pave the way for new perspectives in the search for a therapeutic approach that can target the primary defect, or compensate for it by by-passing the primary defect.

However, the models available so far still limit our comprehension about PHOX2B role in the development of ANS and in the pathogenesis of CCHS. Immortalized cell lines may not recapitulate the physiology of a normal cell or do not have the genetic background of cells from human patients because of their genetic and metabolic defects. Although animal models are very useful to study autonomic circuits, connectivity, regulation, and related disorders, they have significant limitations due to the distinct physiologic mechanisms between rodents and humans. Postmortem tissue analysis is a great tool for studying cellular defects at the end of the disease, but shows some limitations for studying the molecular and biochemical pathophysiology of autonomic neurons. Moreover, in the case of CCHS, the availability of postmortem tissues is limited due to the rarity of the disease. Patient blood samples are also available to study ANS disorders, and recently in CCHS this has enabled to identify an increased oxidative stress in this rare disease (Degl’Innocenti et al., 2018). However, the use of peripheral tissues for studying disease mechanisms and developing treatment options in CCHS could be a problem, due to the fact that the disease does not manifest in blood tissue. The development of methods to efficiently obtain neurons from induced-pluripotent stem cells, obtained by reprogramming somatic cells of patients with a certain disease, is a challenging opportunity to investigate at the molecular level the developmental defect, in the same genomic background of the patient.

### The Disease in a Dish: The Induced Pluripotent Stem Cells (iPSCs) as a New Tool to Study CCHS Pathogenesis

Neurons obtained by differentiating human pluripotent stem cells (hPSCs), including embryonic stem or induced pluripotent stem cells (hESCs/hiPSCs), are a promising model for studying human neural development and disease (Avior et al., 2016; Pacitti et al., 2019), including rare pediatric disorders (Freel et al., 2020). Compared to hESCs, iPSCs can be obtained by reprogramming somatic cells (such as adult skin cells) from individual patients. Therefore, the iPSCs derived from fibroblasts of CCHS patients offer a new opportunity to obtain neuronal cells that carry the different mutations and to capture the entire patient’s genetic profile, including all of the genetic modifiers that have important, yet largely unknown, roles in the pathology of CCHS (Bellin et al., 2012). This technology has previously been used to model familial dysautonomia (which, like CCHS, is a genetic disease related to ANS dysfunction) and has provided evidence that iPSCs can recapitulate disease-related phenotypes and are useful for studying molecular defects (Lee et al., 2009; Lee and Studer, 2011; Zeltner et al., 2016). Recently, it has been reported the generation of iPSC lines from two CCHS identical twins with *PHOX2B* PARM mutation (20/25 genotype). Both lines express pluripotency markers and can differentiate into the three germ layers, but the possibility to use these iPSCs to recapitulate aspects of CCHS has not yet been fully investigated (Falik et al., 2020).

While significant progress has been made on the generation and utilization of CNS cell types from hPSCs, very few studies have been reported on the derivation of the neurons affected in CCHS [neural crest derived neurons and CNS neural lineages, such as central noradrenergic neurons (NANs), and hindbrain branchio motor (bMNs) and visceral motor neurons (vMNs)].

Reported methods of generating NANs and vMNs from pluripotent stem cells rely on the over-expression of *PHOX2B* (or alternatively *PHOX2A* for vMNs and bMNs) (Panman et al., 2011; Mong et al., 2014; De Santis et al., 2018).

Peripheral neurons can be generated from iPSCs using a multistage differentiation protocol that starts with the generation of neural crest (NC) precursors, as reported with the differentiation of NC cells from hESCs (Lee et al., 2007). This differentiation protocol has been modified and improved to efficiently obtain a higher number of pure PHOX2B^+^ neurons (Chambers et al., 2009, 2013; Lee et al., 2010; Menendez et al., 2013; Huang et al., 2016; Oh et al., 2016; Zeltner et al., 2016; Frith et al., 2018; Kirino et al., 2018). ANS disorders, such as familial dysautonomia (Zeltner et al., 2016) and Hirschsprung’s disease have been modeled by using hPSC (Workman et al., 2017). Interestingly, mutated enteric NC derived from hPSCs carrying a typical HD patients *PHOX2B* mutation showed a defect in migration and differentiation in intestinal organoids.

The setting up of *in vitro* protocols to obtain differentiated neurons relevant to CCHS is an ambitious task; one of the major challenges is in generating well-characterized, mature and functional cell types. Oh et al. (2016) use co-cultures of hPSCs-derived sympathetic neurons with cardiomyocytes to overcome this issue and to enhance the maturity of the neurons and improve functionality.

Despite the fact that the use of hPSC technology for CCHS modeling is still in its infancy its great potential to provide new tools for investigating CCHS pathogenesis and validating drugs to ameliorate CCHS symptoms is widely acknowledged in the scientific community.

## Author Contributions

SD and RB contributed equally to the conceptualization and to the writing of the manuscript. SC contributed to writing and reviewing. DF revised and edited the final draft of the manuscript. All authors contributed to the article and approved the submitted version.

## Conflict of Interest

The authors declare that the research was conducted in the absence of any commercial or financial relationships that could be construed as a potential conflict of interest.
